# Dr. James E. Pilcher, President of the Fifth Censorial District Medical Association of Pennsylvania

**Published:** 1907-09

**Authors:** 


### Dr. James E. Pilcher, President of the Fifth Censorial District Medical Association of Pennsylvania.

AT the second annual meeting of the Fifth Censorial District Medical Association, of Pennsylvania, held at Mount Holly Park, Pa., on August 13, the district comprising the counties of Adams, Cumberland, Franklin, Fulton and York, Major James Evelyn Pilcher, editor of the *Military Surgeon,* the world's authority on military medicine, was elected president. The honor conferred on Dr. Pilcher is well deserved for he has labored unceasingly for the success of the organisation which has chosen him as its executive head. His well known activity will give the association an administration which will place it in the front rank of Pennsylvania medical societies. Dr. Pilcher's address on taking the chair was characteristic. He accepted the office and proceeded to a speech of welcome which was delivered in that quaint and open face style of oratory for which he is famous. He figuratively opened his arms and gathered in the whole medical profession; and he had not been talking two minutes before the members of the association realised that they had not only elected a brilliant man as their president, but one who would make the organisation an instrument for the betterment of the profession in the district. Dr. Pilcher's administration will be one of satisfying success and the JOURNAL congratulates the Fifth Censorial District Medical Association on selecting him as its president.

